# Improving specificity of *Bordetella pertussis* detection using a four target real-time PCR

**DOI:** 10.1371/journal.pone.0175587

**Published:** 2017-04-12

**Authors:** Helena Martini, Liselot Detemmerman, Oriane Soetens, Erlangga Yusuf, Denis Piérard

**Affiliations:** Vrije Universiteit Brussel (VUB), Universitair Ziekenhuis Brussel (UZ Brussel), Department of Microbiology and Infection Control and Belgian National Reference Centre for Bordetella Pertussis, Brussels, Belgium; Universidad Nacional de la Plata, ARGENTINA

## Abstract

The incidence of whooping cough, a contagious respiratory disease caused by *Bordetella pertussis*, is on the rise despite existing vaccination programmes. Similar, though usually milder, respiratory symptoms may be caused by other members of the *Bordetella* genus: *B*. *parapertussis*, *B*. *holmesii*, and *B*. *bronchiseptica*. Pertussis diagnosis is mostly done using PCR, but the use of multiple targets is necessary in order to differentiate the different *Bordetella* spp. with sufficient sensitivity and specificity. In this study we evaluate a multiplex PCR assay for the differentiation of *B*. *pertussis* from other *Bordetella* spp., using the targets IS*481*, IS*1001*, IS*1002*, and *recA*. Moreover, we retrospectively explore the epidemiology of *Bordetella* spp. infections in Belgium, using the aforementioned assay over a three-year period, from 2013 until 2015.

## Introduction

Whooping cough or pertussis is a contagious, acute respiratory illness caused by the Gram-negative coccobacillus *Bordetella pertussis* [1]. It is characterised by a paroxysmal cough, although symptoms can be atypical in infants younger than five months, and even fatal [2]. Pertussis in adolescents and adults is typically milder and presents with less severe symptoms [3], but is still of particular concern due to the associated risk of contagion [4]. Despite existing vaccination programmes, the number of whooping cough cases is still rising in Europe [5, 6] and North America [7].

Besides *B*. *pertussis*, three other members of the *Bordetella* genus are associated with respiratory infection in humans: *B*. *parapertussis*, *B*. *holmesii*, and to a lesser degree *B*. *bronchiseptica* [8]. These *Bordetella* species can cause respiratory disease with nonspecific symptoms as well as symptoms similar to those caused by *B*. *pertussis*, but milder [9]. However, pertussis vaccines do not provide cross-protection against these related *Bordetella* spp. [10, 11] Therefore, the differentiation of *B*. *pertussis* from other *Bordetella* spp. in respiratory illness is important for the evaluation of the vaccines’ efficacy, as misdiagnosis can lead to misinterpretation of the vaccines’ failure rate. Moreover, accurate identification of *B*. *pertussis* is needed to determine the necessity of prophylactic antibiotic treatment for the index cases and their contacts, as its utility in *B*. *parapertussis* and *B*. *holmesii* cases is still unclear [12, 13, 14].

As culture is difficult and lacks sensitivity, the diagnosis of *Bordetella* spp. infections often involves real-time polymerase chain reaction (PCR) of nasopharyngeal specimens, which is much more sensitive than bacterial culturing [15, 16, 17]. Segments of insertion sequences (IS) are often used as targets, particularly IS*481* and IS*1001* for *B*. *pertussis* and *B*. *parapertussis* respectively. Tests using these targets are sensitive because the IS are present in numerous copies in the bacterial genomes, but they are not species-specific. IS*481* is found in *B*. *pertussis*, *B*. *holmesii*, and some *B*. *bronchiseptica* [16]. IS*1001* is found in *B*. *parapertussis* and some *B*. *bronchiseptica* [17]. While hIS*1001*, which is found in *B*. *holmesii*, is highly homologous to IS*1001*, there are some sequence differences [17]. Thus, IS*481* and IS*1001* can be used as targets for screening, but confirmation with other targets will improve specificity.

IS*1002* and *recA* have been used previously as secondary targets [18, 19]. IS*1002* is found in *B*. *pertussis*, *B*. *parapertussis*, and some *B*. *bronchiseptica*, while *recA* is found in *B*. *holmesii*. Other *Bordetella* spp. also carry *recA*, but several polymorphisms allow for specific targeting [18]. When combined, the targets IS*481*, IS*1001*, IS*1002*, and *recA* can be used to differentiate *Bordetella* spp. in respiratory illness. IS*481* and IS*1001* can be used to screen for *B*. *pertussis* and *B*. *parapertussis* respectively, while IS*1002* can confirm a positive screen, or alternatively, absence of IS*1002* and presence of *recA* confirms a case of *B*. *holmesii*.

Most commercially available diagnostic PCR assays for pertussis use fewer targets and do not differentiate between *B*. *pertussis* and other *Bordetella* spp. such as *B*. *holmesii*. In order to improve specificity, we evaluate the use of the four PCR targets IS*481*, IS*1001*, IS*1002*, and *recA*, in the differentiation of *B*. *pertussis* from other *Bordetella* spp. that cause respiratory illness. Furthermore, we describe the incidence of respiratory infections caused by *Bordetella* spp. in Belgium from 2013 until 2015 using the described assay.

## Materials and methods

### Clinical sample preparation

Prior to nucleic acid (NA) extraction, viscous samples were pre-treated with Sputasol (Oxoid Ltd., Basingstoke, England) in a 1:1 dilution. 100 μl of each sample was used for NA extraction using NucliSENS easyMag (bioMérieux, Grenoble, France) according to the manufacturer’s instructions. Phocine herpesvirus (PhHV) was added as an internal control to monitor the extraction and the amplification’s efficiency [20].

### Bacterial strain preparation

Reference strains as well as previously well-defined strains from clinical isolates were stored at -80°C prior to use, then grown on laboratory-prepared Regan-Lowe agar plates (Oxoid Ltd., Basingstoke, England) at 35°C. One colony of each strain was suspended in 200 μl Tris-EDTA (pH 8) and boiled to obtain the template NA.

### Real-time PCR

Primers and probes for IS*481*, IS*1001*, and IS*1002* were based on Roorda et al. (2011) [19], those for *recA* were based on Guthrie et al. (2010) [18], and those used for internal control were based on van Doornum et al. (2003) [21] (sequences in Table 1). Primers and probes were manufactured by Eurogentec (Liège, Belgium), MGB probes were manufactured by Applied Biosystems (Warrington, UK).

**Table 1:** Sequences and labelling of real-time PCR primers and probes.

Target	Primer/probe	Sequence (5’-3’) and labels
IS*481*	Forward primer	GCCGGATGAACACCCATAAG [19]
	Reverse primer	GCGATCAATTGCTGGACCAT [19]
	Probe	6FAM-CGATTGACCTTCCTACGTC-MGBNFQ [19]
IS*1001*	Forward primer	AATTGCTGCAAGCCAACCA [19]
	Reverse primer	CCAGAGCCGTTTGAGTTCGT [19]
	Probe	VIC-ACATAGACCGTCAGCAG-MGBNFQ [19]
IS*1002*	Forward primer	CTAGGTCGAGCCCTTCTTGTTAAC [19]
	Reverse primer	GCGGGCAAGCCACTTGTA [19]
	Probe	HEX-CATCGTCCAGTTCTGTTGCATCACCC-BHQ1 [19]
*recA*	Forward primer	CGGTTCGCTGGGTCTCG [18]
	Reverse primer	CCCGCGGCAGACCAC [18]
	Probe	6FAM-CATCGCATTGGGCG-MGBNFQ [18]
PhHV	Forward primer	GGGCGAATCACAGATTGAATC [21]
	Reverse primer	GCGGTTCCAAACGTACCAA [21]
	Probe	CY5-TTTTTATGTGTCCGCCACCATCTGGATC-BHQ2 [21]

**Fig 1:**
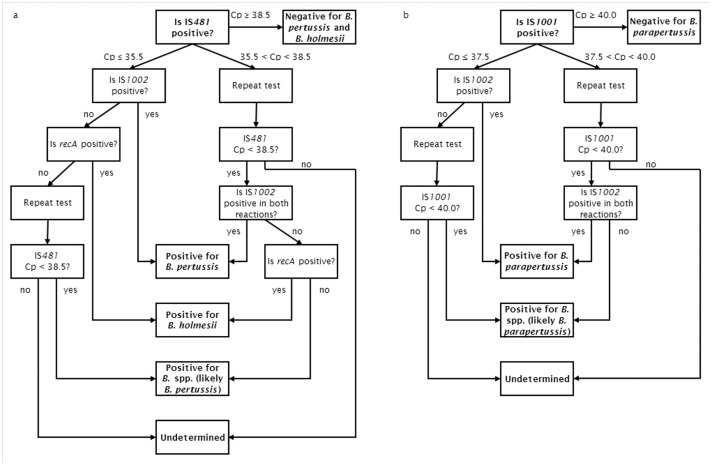
Algorithm for the interpretation of the four-target real-time PCR assay for the detection of *Bordetella* for samples positive for IS*481* (a) and IS*1001* (b).

Two different multiplex PCR’s were performed, one for the detection of IS*481* and IS*1001*, and one for the detection of *recA* and IS*1002*. A 50 μl reaction mixture consisting of 0.3 μM of each of the primers, 0.2 μM of each of the probes, iQ Multiplex Powermix (Bio-Rad Laboratories, Temse, Belgium), and 5.0 μl of the extracted NA was used in each PCR. Amplification was performed on the LightCycler 480 II PCR system (Roche Diagnostics, Mannheim, Germany). The PCR thermal profile consisted of a 3-minute cycle at 95°C, followed by 45 cycles of 15 seconds at 95°C and 1 minute at 60°C. Acquisition of the fluorescence signal was set during each cycle. A final cooling step of 30 seconds at 40°C was included. The crossing point (Cp) values were determined automatically using the “second derivative maximum” method present in the Roche LightCycler 480 Software, version 1.5. An algorithm with cut-off values was developed and used for the interpretation of the real-time PCR results (Fig 1).

### Bacterial culture

For culture, clinical specimens were inoculated onto laboratory-prepared Regan-Lowe agar upon arrival at the facilities. Plates were incubated at 35°C in a humidified aerobic atmosphere and examined daily for suspect colonies for up to twelve days.

Suspect colonies were identified using the microflex LT MALDI-TOF platform and MALDI Biotyper 3.0 software (Bruker Daltonics GmbH, Leipzig, Germany) [22, 23]. Identification to the species level was accepted when all matches with a log score above 1.7 belonged to the same species, or when the best match had a log score both above 2.0 and more than 0.200 higher than that of the other matched species.

When MALDI-TOF results were inconclusive, the species was determined based on biochemical characteristics: growth or absence of growth on charcoal agar, Haemophilus agar and MacConkey agar, presence of oxidase and presence of urease. Positive results for all characteristics indicated *Bordetella bronchiseptica*. Growth on charcoal agar and Haemophilus agar and presence of urease but not oxidase indicated *Bordetella parapertussis*. Growth on charcoal agar only, combined with the presence of oxidase but not urease indicated *Bordetella pertussis* [24].

### Accuracy of real-time PCR

Accuracy was verified using a group of selected, previously well characterised strains derived from clinical samples, as well as reference strains (ATCC9340, ATCC9797, LMG15945, LMG15946), samples from Quality Control for Molecular Diagnostics (QCMD) 2011, and the extracted NA of different strains from a EUpert-labnet network panel (http://www.ecdc.europa.eu/en/publications/Publications/20120906-TER-EQA-pertussis.pdf). These samples included twenty-seven *B*. *pertussis* samples, nineteen *B*. *parapertussis* samples, seven *B*. *holmesii* samples, four samples of other *Bordetella* spp. and five non-*Bordetella* samples.

### Analytical specificity of real-time PCR

The specificity of the primers and probes was first checked by performing a BLAST search on their sequences (http://blast.ncbi.nlm.nih.gov/Blast.cgi). Specificity was further tested by performing the previously described real-time PCR on twelve well-defined *B*. *bronchiseptica* strains and seven *B*. *holmesii* strains, as well as other *Bordetella* spp. (*B*. *avium* (n = 2), *B*. *petrii* (n = 2), *B*. *hinzii* (n = 4), *B*. *trematum* (n = 2), *B*. *ansorpii* (n = 2)). The following bacteria of different genera were also tested: *Acinetobacter baumani* (n = 1), *A*. *iwofii* (n = 1), different *Burkholderia* spp. (n = 20), *Enterobacter aerogenes* (n = 2), *Haemophilus influenzae* (n = 2), *Klebsiella pneumoniae* (n = 1), *Moraxella catarrhalis* (n = 1), *Neisseria meningitides* (n = 1), *Pseudomonas aeruginosa* (n = 2), *P*. *putida* (n = 2), *Staphylococcus aureus* (n = 2), *Stenotrophomonas maltophilia* (n = 1), and different *Streptococcus* spp. (n = 8).

### Analytical sensitivity of real-time PCR

Analytical sensitivity was determined using three reference strains: ATCC9797 (*B*. *pertussis*), ATCC15311 (*B*. *parapertussis*), and LMG15945 (*B*. *holmesii*). They were suspended in physiological saline at a theoretical concentration of 1.5 × 10^8^ CFU/ml (0.5 McFarland). Serial dilutions were made from these suspensions, and 100-μl aliquots of each dilution were extracted and tested with real-time PCR. Tests were performed ten to twelve times for each strain. The analytical sensitivity was determined by calculating the limit of detection (LOD 95%), i.e. the lowest concentration that is detected 95% of the time, using probit regression analysis in SPSS Statistics 20.0 (IBM Corp., Armonk, NY, USA).

### Precision of real-time PCR

Replication experiments were performed to determine precision, using three dilutions (strong positive, positive and weak positive) of the reference strains in physiological water. These samples were tested at least ten times in five different runs over the course of five days. The intra-run and inter-run coefficients of variance (CV) were calculated based on the Cp value of the assay using SPSS Statistics 20.0.

### Laboratory-based surveillance of pertussis

Surveillance data on circulating *B*. *pertussis*, *B*. *parapertussis* and *B*. *holmesii* in Belgium, based on the results of the real-time PCR for IS*481*, IS*1001*, *recA*, and IS*1002*, were gathered retrospectively from 11919 respiratory samples collected from January 2013 until December 2015.

## Results

### Accuracy of real-time PCR

All clinical strains and reference strains were identified correctly: all *B*. *pertussis* strains were positive for IS*481* and IS*1002*, and negative for *recA* and IS*1001*. All *B*. *parapertussis* strains were positive for IS*1001* and IS*1002*, and negative for *recA* and IS*481*. All *B*. *holmesii* strains were positive for IS*481* and *recA*, and negative for IS*1001* and IS*1002*.

Out of the twelve QCMD samples, all but one sample tested correctly: four out of five *B*. *pertussis* strains were positive for IS*481* and IS*1002*, and negative for *recA* and IS*1001*, only one very weak positive (10 CFU/ml) *B*. *pertussis* sample was negative for all targets. The *B*. *parapertussis* strain was positive for IS*1001* and IS*1002*, and negative for *recA* and IS*481*. The *B*. *holmesii* strain was positive for IS*481* and *recA*, and negative for IS*1001* and IS*1002*. The *B*. *bronchiseptica*, *B*. *hinzii*, and non-*Bordetella* samples tested negative for all targets.

Finally, out of ten NA samples from the EUpert-labnet network panel, eight were identified correctly. One *B*. *pertussis* NA sample with a low concentration of 0.02 pg/μl was negative for IS*1002*, but positive for IS*481*. One *B*. *holmesii* sample was positive for IS1001 as well as for IS481 and recA. Using the algorithm developed for our assay, these results did not lead to an incorrect identification (S1 Table).

### Analytical specificity of real-time PCR

BLAST analysis showed that IS*481* and IS*1001* may cross-react with *B*. *bronchiseptica* and *B*. *holmesii*. IS*1002* was specific for *B*. *pertussis* and *B*. *parapertussis*. Finally, *recA* showed cross-reactivity with some *Burkholderia* spp.

Following BLAST analysis the assay was performed on different strains. Six out of seven *B*. *holmesii* strains were negative for both IS*1001* and IS*1002*, one was positive for IS*1001*. Out of twelve tested *B*. *bronchiseptica* strains, four were positive for IS*481* and two for IS*1001*. One IS*481*-positive strain was also positive for IS*1002*, and as such could have been misinterpreted as *B*. *pertussis*. No cross-reaction was detected in other *Bordetella* spp.

As BLAST analysis suggested possible cross-reactivity of *recA* with some *Burkholderia* spp., this target was tested in twenty *Burkholderia* strains, belonging to eleven different species. Cross-reaction was detected in one *Burkholderia stabilis* strain and one *Burkholderia multivorans* strain. No cross-reaction with any of the other targets was found.

Finally, the assay was tested with various other bacteria, including bacteria that cause respiratory infections as well as commensals. Minor cross-reactivity was found in *Stenotrophomonas maltophilia* for IS*481* and in *Enterobacter aerogenes* for IS1001, however, the other targets remained negative (S1 Table).

### Analytical sensitivity of real-time PCR

The limit of detection (LOD 95%) for IS*481* was 145 CFU/ml for *B*. *pertussis* and 152 CFU/ml for *B*. *holmesii*. For IS*1001*, the LOD 95% was 242 CFU/ml for *B*. *parapertussis*. The *recA* assay had an LOD 95% of 2577 CFU/ml for *B*. *holmesii*. Finally, for IS*1002* the LOD 95% was 1954 CFU/ml for *B*. *pertussis* and 1725 CFU/ml for *B*. *parapertussis* (S1 Table).

### Precision of real-time PCR

The coefficients of variance (CV) for the detection of IS*481* ranged from 1.1% to 8.4% intra-run, and from 1.8 to 5.4% inter-run. For IS*1001*, they ranged from 1.7% to 3.7% intra-run, and from 0.8% to 2.0% inter-run. The intra-run CV for *recA* ranged from 1.8% to 2.4%, and the inter-run CV ranged from 0.8% to 1.2%. Finally, for IS*1002*, the intra-run and inter-run CV ranged from 1.9% to 4.0% and from 0.3% to 1.9% respectively for *B*. *pertussis*, and from 2.3% to 6.2% and from 1.3% to 3.8% respectively for *B*. *parapertussis* (S1 Table).

### Laboratory-based surveillance of pertussis

The Belgian National Reference Centre (NRC) for *B*. *pertussis* performed real-time PCR on 11919 respiratory samples between 2013 and 2015. These data were interpreted using the algorithm presented above (Fig 1). Among these samples, 1189 (10.0%) were found positive for *B*. *pertussis*. 872 (73.3%) of these were positive for both IS*481* and IS*1002* and reported “positive for *B*. *pertussis*”, while 317 (26.7%) were positive for IS*481* only and reported as “positive for *Bordetella* spp., probably *B*. *pertussis*”. 151 samples (1.3%) were reported positive for *B*. *parapertussis*, of which 112 (74.2%) were positive for both IS*1001* and IS*1002* (reported “positive for *B*. *parapertussis*”), and 39 (25.8%) positive for IS*1001* only (reported as “positive for *Bordetella* spp., probably *B*. *parapertussis*”). Furthermore, 15 samples (0.1%) were found positive for *B*. *holmesii*, and 425 samples (3.6%) were reported as “undetermined”, i.e. they tested positive for IS*481* or IS*1001* but negative for *IS1002* and *recA*, then negative for all targets upon repeat testing (S2 Table).

Of the samples found positive for *B*. *pertussis* with real-time PCR, 28.8% were also positive in culture. For *B*. *parapertussis*, this percentage was similar at 29.8%. Only two of the samples reported as “positive for *Bordetella* spp., probably *B*. *pertussis*” and none of the samples reported as “positive for *Bordetella* spp., probably *B*. *parapertussis*” were recovered in culture. These samples were associated with higher Cp values. The association between Cp value and culture recovery is presented in Table 2. Out of the fifteen *B*. *holmesii* samples, five were found positive in culture.

**Table 2:** Association between Cp value and recovery in culture, for samples positive for IS481 (a) and IS1001 (b).

a	**Cp value IS481**	**PCR positive**	**Culture positive (B. pertussis)**	**Recovery culture/PCR**
	**Cp ≤ 20**	165	133	80.6%
	**20 < Cp ≤ 25**	122	81	66.4%
	**25 < Cp ≤ 30**	227	89	39.2%
	**Cp > 30**	675	39	5.8%
	**Total**	1189	342	28.8%
b	**Cp value IS1001**	**PCR positive**	**Culture positive (B. parapertussis)**	**Recovery culture/PCR**
	**Cp ≤ 20**	7	7	100.0%
	**20 < Cp ≤ 25**	16	13	81.3%
	**25 < Cp ≤ 30**	22	14	63.6%
	**Cp > 30**	105	11	10.5%
	**Total**	151	45	29.8%

A few samples were found positive for more than two targets. Seven samples were positive for IS*481*, IS*1001* and IS*1002*, and were interpreted as co-infection of *B*. *pertussis* and *B*. *parapertussis*. In culture, two of these were found positive for *B*. *pertussis* and three for *B*. *parapertussis*. Two other samples were positive for IS*481*, *recA* and IS*1002* and were interpreted as co-infection of *B*. *pertussis* and *B*. *holmesii*. These samples were negative in culture.

## Discussion

In this study, the use of a multiplex real-time PCR assay for the detection of *B*. *pertussis*, *B*. *parapertussis*, and *B*. *holmesii* was investigated. By combining a sensitive screening method with a confirmation assay, the risk of false positive results is reduced and the specificity increased. The strength of our study lies in the extensive testing of the real-time PCR targets, during validation and during a three-year study with clinical samples.

Our real-time PCR assay uses the targets IS*481*/IS*1001* for screening and *recA*/IS*1002* for confirmation. We showed that this method is accurate, sensitive, fairly specific, and precise. *B*. *pertussis*, *B*. *parapertussis*, and *B*. *holmesii* reference and clinical strains were identified correctly. The detection limit of our method was in line with that of other assays described for IS*481* and IS*1001*, ranging from 145 to 242 CFU/ml for *B*. *pertussis*, *B*. *parapertussis* and *B*. *holmesii* [19, 25, 26, 27]. We also found that the sensitivity of the PCR for *recA*/IS*1002* is around ten times lower than that of the PCR for IS*481*/IS*1001*. This lower sensitivity can be explained by the lower copy numbers of *recA* and IS*1002* genes.

The targets used in this study were fairly specific. The targets of the screening assay, IS*481* and IS*1001*, do not show cross-reactivity with other respiratory pathogens. However, they do show cross-reactivity with *B*. *holmesii* and sometimes with *B*. *bronchiseptica*. IS*481*, IS*1001* and IS*1002* have all been previously detected in some *B*. *bronchiseptica* strains [19]. Therefore the possibility of cross-reactivity should be taken into account when using these targets. This problem is limited by the use of target combinations: both IS*481* (screening) and IS*1002* (confirmation) need to be positive to identify *B*. *pertussis*, both IS*1001* and IS*1002* need to be positive to identify *B*. *parapertussis*.

Using our method, about a fourth of the cases positive for IS*481* remained negative for IS*1002* (and *recA*). These cases are reported as “positive for *Bordetella* spp., probably *B*. *pertussis*”. Samples positive for IS*1001* but negative for IS*1002* (and *recA*) are reported as “positive for *Bordetella* spp., probably *B*. *parapertussis*”. Cases such as these occur relatively frequently due to the difference in sensitivity between the screening and the confirmation targets, the latter being present in much lower copy numbers.

For weak positive results (i.e. high Cp values) in the screening assay, cross-reactivity, in particular with *B*. *bronchiseptica*, cannot be ruled out. However, *B*. *bronchiseptica* is rarely found in respiratory samples from patients displaying symptoms of whooping cough: in over 2000 tested samples only one cross-reacting *B*. *bronchiseptica* strain was cultured [28]. Therefore the probability of *B*. *bronchiseptica* causing a false positive result may be considered negligible. Because of the low occurrence of *B*. *holmesii* and *B*. *bronchiseptica* in patients displaying pertussis symptoms, it is acceptable to consider cases with a Cp lower than 38.5 for IS*481* (or lower than 40 for IS*1001*) as probable cases of *B*. *pertussis* (or *B*. *parapertussis*), even if they are negative for IS*1002* and *recA*.

Based on the extensive validation of the real-time PCR, an algorithm was developed for its interpretation (Fig 1). Using high copy number targets such as IS*481* and IS*1001* increases the sensitivity of the assay at the cost of an increased risk of false positives. This risk can be reduced by combining multiple targets, as demonstrated in this study.

The implementation of cut-off values will also reduce false positivity, as shown previously in a quality control study [29], and is recommended by the CDC (http://www.cdc.gov/pertussis/clinical/diagnostic-testing/diagnosis-pcr-bestpractices.html).

In this study, a cut-off value based on the LOD 95%, determined during validation of the assay, was used. Positive results with a Cp value higher than this value should be repeated, and discordant results should be reported to the requesting physician as “undetermined”, to be considered negative or investigated further based on clinical presentation and serology. A second cut-off value to reduce false positivity was implemented as well. Samples with Cp values higher than this cut-off are immediately reported as negative.

During the study period, *B*. *pertussis* was diagnosed about ten times as much as *B*. *parapertussis*, which was diagnosed about ten times as much as *B*. *holmesii*. Less than four percent of the clinical samples could not be reliably identified using our assay (“undetermined”).

By including the target *recA* in the assay, *B*. *holmesii* can be differentiated from *B*. *pertussis*. This is important in routine diagnosis as *B*. *holmesii* is increasingly reported in Europe, and has often been misidentified as *B*. *pertussis* in the past [13, 14, 28]. Using our assay, we found fifteen *B*. *holmesii* strains during the study period, suggesting that *B*. *holmesii* might indeed occur more frequently than previously reported in Belgium [28].

The algorithm was not developed for the interpretation of co-infections, and they are difficult to identify with the presented method. However, co-infection is likely if the assay is positive for more than two targets. Several possible co-infections were detected during the study that could not be confirmed by culture. Based on the large amount of samples, it seems that the likelihood of co-infections is limited (less than 0.1%).

We demonstrated in this study that there is a high concordance between the Cp value of a sample and the probability of recovery in culture. This finding is consistent with previous results [30]. As culturing of *B*. *pertussis* is no longer performed diagnostically, but rather for the acquiring of strains to be used in epidemiologic typing and research, it may be worthwhile to try to culture only samples with low Cp values. For *B*. *pertussis*, culturing has a high success rate for Cp values up to 25. For Cp values between 25 and 30, this rate drops to less than 50%. Culturing in these cases should still be considered for laboratories with a particular interest in obtaining as many strains as possible for epidemiological and virulence studies. When Cp values are higher than 30 the success rate becomes negligible (less than 6%) and culturing is not advisable.

Several PCR targets specific to *B*. *pertussis* have been described in literature, such as the pertussis toxin promoter (*ptxP*), recommended by the Pertussis PCR Consensus Group [31]. However, the sensitivity of real-time PCR with *ptxP* as a target is considerably lower than with IS*481* [32]. Other possible targets for *B*. *pertussis*, such as the pertactin genes, BP*283*, BP*485*, and *ptxS1* have also been described, but often show cross-reactivity with other *Bordetella* spp. [33, 34] Recently an assay with a porin protein as target was described as specific for *B*. *pertussis*, but a larger specificity study would be needed to confirm this [32].

In conclusion, multiplex real-time PCR with targets IS*481*/IS*1001* can be used for screening, followed by confirmation with targets *recA*/IS*1002*, in order to differentiate *B*. *pertussis* from other *Bordetella* spp. that cause respiratory infection or colonisation.

## Supporting information

S1 Table Results used to determine accuracy, specificity, analytical sensitivity and precision. (XLSX)

S2 Table Clinical results from January 2013 until December 2015. (XLSX)
